# High-index-contrast photonic structures: a versatile platform for photon manipulation

**DOI:** 10.1038/s41377-022-01021-1

**Published:** 2022-11-01

**Authors:** Young-Bin Kim, Jin-Woo Cho, Yun-Jo Lee, Dukkyu Bae, Sun-Kyung Kim

**Affiliations:** 1 Department of Applied Physics, Kyung Hee University, Yongin, Gyeonggi-do 17104 Republic of Korea; 2 Hexa Solution Co., Ltd, Suwon, Gyeonggi-do 16229 Republic of Korea

**Keywords:** Optoelectronic devices and components, Inorganic LEDs, Photonic crystals

## Abstract

In optics, the refractive index of a material and its spatial distribution determine the characteristics of light propagation. Therefore, exploring both low- and high-index materials/structures is an important consideration in this regard. Hollow cavities, which are defined as low-index bases, exhibit a variety of unusual or even unexplored optical characteristics and are used in numerous functionalities including diffraction gratings, localised optical antennas and low-loss resonators. In this report, we discuss the fabrication of hollow cavities of various sizes (0.2–5 μm in diameter) that are supported by conformal dielectric/metal shells, as well as their specific applications in the ultraviolet (photodetectors), visible (light-emitting diodes, solar cells and metalenses), near-infrared (thermophotovoltaics) and mid-infrared (radiative coolers) regions. Our findings demonstrate that hollow cavities tailored to specific spectra and applications can serve as versatile optical platforms to address the limitations of current optoelectronic devices. Furthermore, hollow cavity embedded structures are highly elastic and can minimise the thermal stress caused by high temperatures. As such, future applications will likely include high-temperature devices such as thermophotovoltaics and concentrator photovoltaics.

## Abstract (web summary)

This paper reviews the fabrication and functionalities of high-refractive-index-contrast optical structures that can serve as versatile optical platforms to address the limitations of current optoelectronic devices.

## Graphical abstract

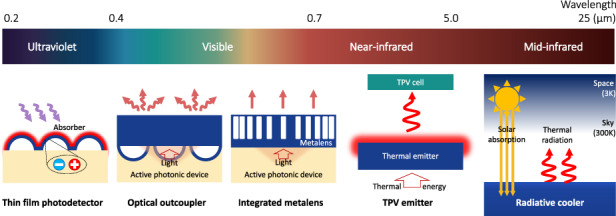

## Introduction

Increasing the refractive index contrast (Δ*n*) of the components of an optoelectronic device can improve its optical functionality^1–3^. This assertion can be verified by calculating the diffraction strength of a grating with a sinusoidal variation of the refractive index *n*(*r*), which is given by:

1 $$n\left( r \right) = n_0 + \Delta n\,\cos \left( {\vec k \cdot \vec r} \right)$$

where *n*_0_ and *k* are the average refractive index and the propagation vector of the grating, respectively. The electric field of the diffracted light $E\left( {\vec r} \right)$ is then obtained by applying Bloch’s theorem, which is expressed as

2 $$E\left( {\vec r} \right) = A\left( {\vec r} \right)e^{ - i\vec k_{inc} \cdot \vec r} = An_0e^{ - i\vec k_{inc} \cdot \vec r} + \frac{{A\Delta n}}{2}e^{ - i\left( {\vec k_{inc} - \vec \kappa } \right) \cdot \vec r} + \frac{{A\Delta n}}{2}e^{ - i\left( {\vec k_{inc} + \vec \kappa } \right) \cdot \vec r}$$

where $A\left( {\vec r} \right)$ is a periodic function with the same periodicity as *n*(*r*) and $\vec k_{inc}$ is the wave vector of the incident light. For the right-hand side of Eq. (2), the second and third terms indicate the first-order diffraction modes, for which their amplitudes and Δ*n* have a linear relationship. Therefore, a void (i.e. *n* = 1) grating imparts maximum diffraction strength if it is embedded in a high-*n* medium. As a result, a void, also known as an air gap or hollow cavity (HC), produces a variety of enhanced or even unexplored optical features, particularly when combined with a high-*n* ambient medium. The term ‘cavity’ technically refers to an optical structure supporting resonant modes. Nevertheless, any hollow morphology, including air voids and gaps, will also be referred to as HC, as discussed below, for the sake of convenience.

**Fig. 1:**
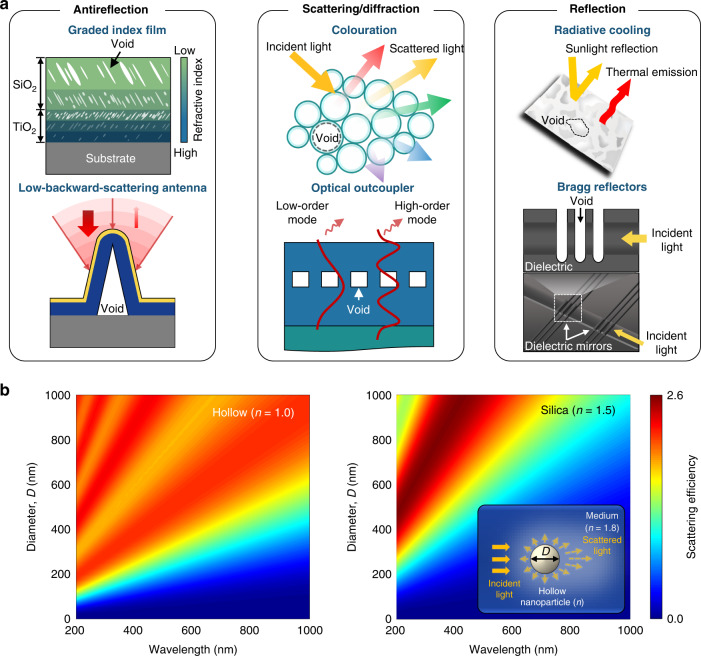
HCs for advanced optical functionalities. **a** HCs with a variety of optical functionalities: (left) antireflection^7^, (middle) scattering/diffraction^6,8^ and (right) reflection^10,11^. **b** Scattering efficiency spectra of (left) hollow (*n* = 1.0) and (right) silica (*n* = 1.5) nanoparticle as a function of *D* of the nanoparticle. Inset: Schematic of light scattering from a hollow nanoparticle embedded in a relatively high-*n* medium (*n* = 1.8)

An individual HC or an array of HCs can be exploited as a localised optical antenna^4^, a low-loss resonator^5^ or a strong-diffraction grating^6^, depending on the scale of the HC relative to the working spectrum. The range of applications can be categorised by function: antireflective films for lenses and solar cells^7^, strong scattering particles for colouration^8^, strong diffraction gratings for light-emitting diodes (LEDs)^9^ and high-reflectivity radiative coolers^5^ and mirrors for lasers^10^ (Fig. 1a). For example, Xi et al. reported that incorporation of voids much smaller than considered wavelengths into dielectrics (e.g. SiO_2_ and TiO_2_) suppressed the surface reflection by adjusting their refractive indices^7^. Kim et al. demonstrated angle-insensitive structural colouration by embedding monodisperse HC particles in a solid matrix^8^. The average spacing between the embedded HCs was readily tuned by adjusting their shell thickness, and the resulting colour was altered over the visible spectrum. Mandal et al. also created hierarchically designed polymers with micro- and nanoscale voids that efficiently backscattered sunlight at wavelengths ranging from the ultraviolet to near-infrared region, corresponding to their sizes^11^. They demonstrated ultrahigh solar reflectance with a near-unity thermal emittance, thus observing a 6 °C drop below the ambient temperature in the daytime. Pruessner et al. constructed high-quality microcavities using periodically arranged quarter-wavelength air gaps and integrated them into silicon waveguides^10^.

In addition, LEDs must minimise the ‘internal’ reflection losses for incident angles (*θ*) above the critical angle to improve their outcoupling efficiencies^12^. To this end, two-dimensional (2D) periodic^9^ or pseudorandom gratings^13^ are essential for extracting trapped photons owing to total internal reflections (TIRs). However, the diffraction strength of the gratings is impeded by Δ*n*, given that the refractive index of the LED medium is fixed. Therefore, there have been few breakthroughs regarding light extraction. Embedding the array of HCs into LED media is an effective way to overcome this index barrier and improve the outcoupling efficiency. The HC grating is also useful for application to directional light sources. For direct-lit LED displays, substrate-side emission must be suppressed because it degrades the image resolution owing to crosstalk between adjacent pixels^14^. The HC grating preferentially excites high-order diffraction modes and can thus steer substrate-side emission into vertical emission through the top faces of LED devices^15^. Likewise, an HC-embedded high-*n* medium can serve as a broadband optical diffuser owing to its strong scattering characteristics, which is evident from the results of electromagnetic simulations (Fig. 1b). In the simulations, to demonstrate the Δ*n* dependent scattering, a single hollow (*n* = 1.0) or silica (*n* = 1.5) nanoparticle was incorporated into a high-*n* medium (*n* = 1.8), and each scattering spectrum was obtained for a variety of diameters (*D*) of the nanoparticle. A comparison of the simulated spectra revealed that even at *D* < 400 nm, the hollow nanoparticles exhibited a high-amplitude scattering over the visible region.

**Fig. 2:**
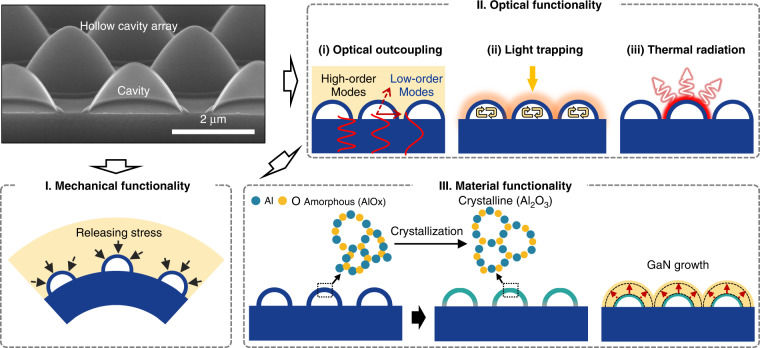
Versatile functionalities of HCs. HCs can serve as multifunctional elements for enhancing the (I) mechanical, (II) optical and (III) material functionalities of optoelectronic devices. (Leftmost, top panel) SEM image of an actual HC array

HCs and their arrays can serve as multifunctional elements that enhance the mechanical and material properties of optoelectronic devices, as well as their optical performance (Fig. 2). In general, HC-embedded structures are highly flexible, thus alleviating the thermal stress triggered by high temperatures^16^. Therefore, their applications can be extended to high-temperature devices, such as thermophotovoltaics and concentrator photovoltaics. Furthermore, HCs that are sustained by a thin (<100 nm) alumina shell can assist in the release of the thermal stress that accumulates during heteroepitaxial GaN growth on a sapphire substrate, resulting in a reduced wafer bow. These mechanical features can improve the internal quantum efficiency of GaN-based LED devices. In addition, the alumina shell acts as a GaN growth template because it is transformed into an α-phase crystallinity during the fabrication of HCs at high temperatures.

**Fig. 3:**
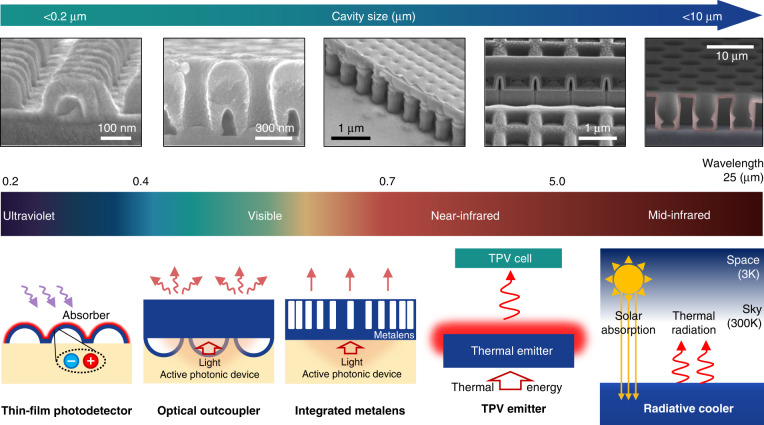
Scalability and applicability of HCs across the electromagnetic spectrum from ultraviolet to mid-infrared wavelengths. The scale of the HCs varies depending on the operating wavelengths. (Top panel) SEM images of HCs of different sizes and (bottom panel) schematic of corresponding applications. SEM images in top panel are reprinted with permission from ref. ^29^ Copyright © 2021 Elsevier, ref. ^4^ Copyright © 2019 and ref. ^5^ Copyright © 2020 American Chemical Society

We emphasise that HCs with appropriate scales can be leveraged for use in a variety of photonic applications with different spectra (Fig. 3), which is indicative of their practical versatility. In the following sections, we review specific HC-based applications: light-emission devices (e.g. LEDs and metalenses), light-absorption devices (e.g. solar cells and photodetectors) and thermal-radiation devices (e.g. thermophotovoltaics and radiative coolers). The morphological parameters (e.g. size, shape, shell material, shell thickness and density in an array) of the HCs are tailored for the intended purpose. Hence, a deep understanding of the fundamental physics regarding the propagation of light and the establishment of generic design principles is important. Furthermore, this knowledge can lead to new insights into a variety of index engineering problems at specific or broad wavelengths.

### Fabrication of HCs

**Fig. 4:**
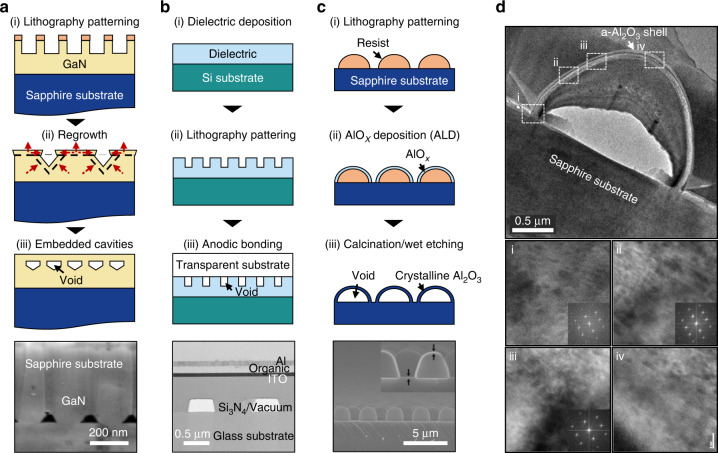
Fabrication of HCs. **a**–**c** (Top panels) Schematic illustrating a fabrication process of **a** epitaxial overgrowth^17^, **b** anodic bonding^21^ and **c** calcination/wet etching^5,22^. (Bottom panels) Scanning electron microscopy (SEM) images of the final structure for each method. **d** (Top panel) Crystallization of alumina shell via calcination process in (**c**). (Bottom panels) High-resolution transmission electron microscope images (labelled as i–iv) at the top panel of the sapphire shell for various regions which are highlighted as four white dashed boxes. Insets: Fast Fourier transformation images at the regions labelled as i–iv. SEM images in (**a**) is reprinted with permission from ref. ^17^ Copyright © 2008 American Institute of Physics, (**b**) from ref. ^21^ Copyright © 2018 Wiley. **c** and **d** are reprinted with permission from ref. ^22^ Copyright © 2015 Elsevier

Various top-down approaches have been employed to fabricate HCs and their arrays. For GaN-based LED devices, the epitaxial overgrowth of GaN on a patterned GaN medium, which is prepared using lithography and etching processes, produces multiple nanoscale voids embedded in the GaN medium^17,18^ (Fig. 4a). This is because of the different GaN growth rates that depend on the crystalline faces. These overgrowth-induced voids improve the outcoupling efficiency because they significantly disturb the waveguide modes that carry photons trapped via TIRs. However, precise control over the morphology of voids is challenging because the initial pattern on the GaN medium tends to deform during overgrowth. This limits the enhancement of the outcoupling efficiency. Furthermore, the addition of manufacturing stages degrades the electrical properties (e.g. leakage current and threshold voltage) of LED devices, resulting in higher fabrication costs^19^. HCs can be embedded in a general dielectric using a wafer-bonding process^20,21^ (Fig. 4b). First, a pattern is defined on a dielectric substrate using standard semiconductor processes. Then, another substrate is bonded to the patterned dielectric substrate using an adhesive gel, by which an array of voids is formed. This method was used to improve the outcoupling efficiencies of organic LEDs by bonding to a transparent glass or polymer substrate. However, wafer-bonding using an adhesive gel is vulnerable to thermal stress and is not suitable for mass production. These HCs in Fig. 4c can be arranged regularly in space and sustained using thin dielectric or metal shells. To fabricate them, a resist was initially patterned using a standard lithography system (e.g. electron-beam lithography, nanoimprinting and a stepper). The pitch, diameter and height of the fabricated HCs were solely determined at the lithography step, whereas their shapes (e.g. cylinder, pyramid, and dome) were controlled to some extent by adopting appropriate thermal reflow conditions after the lithography process. The pitch, diameter (i.e. the base of a dome) and height of the HC array were readily preserved during the thermal reflow process, ensuring their strong diffraction^6^. Then, an alumina shell was deposited to conformally cover the resist pattern using atomic layer deposition (ALD) and removed the inner resist via calcination and/or wet etching. Additional dielectric and metal shells can be deposited with the shell material and thickness varied depending on the intended use.

To iterate, for GaN-based LED devices, an alumina shell was crystallised into the α-phase at high temperatures (typically, >1000 °C) during the calcination step^22^ (Fig. 4d). Therefore, the HCs with a crystalline alumina shell can be simultaneously used as a strong diffraction grating and a pristine GaN growth template. It is noteworthy that the shape of HCs varies by applications with distinct functionalities. For example, a cone is beneficial for light absorption devices such as photovoltaics because they require broadband, antireflecting performance^23^. In comparison, for thermophotovoltaics and radiative coolers (RCs), a cylinder is ideal because it serves as a Fabry-Pérot resonator that can augment light absorption at specific or broad wavelengths^5^. A dome is well suited to the growth template of GaN-based LEDs because GaN grown on a dome-shaped sapphire has fewer dislocations as well as alleviated residual strains, thus improving the internal quantum efficiency^24^.

## Light emission devices

### Optical outcouplers

#### III–V semiconductor light-emitting diodes

**Fig. 5:**
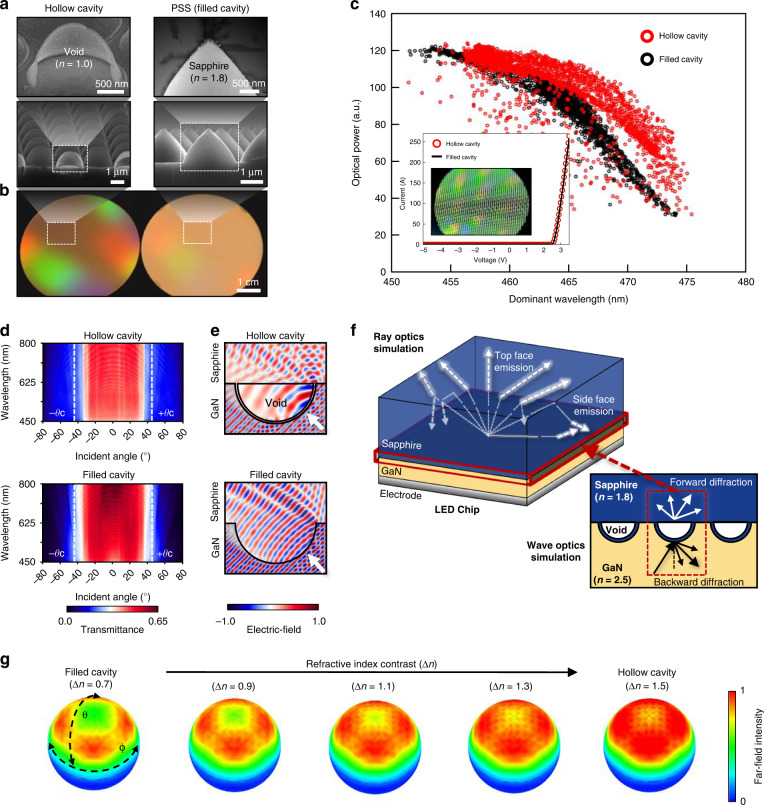
HC-based optical outcoupler for LEDs. **a** Cross-sectional SEM images of the hollow (left) and filled cavities (right). (Top panel) The magnified images exhibit a single cavity structure with a (left) void (*n* = 1.0) and (right) sapphire filled (*n* = 1.8), respectively. **b** Camera images of hollow (left) and filled (right) cavity embedded GaN wafers under white illumination. **c** Probe map data for the optical power recorded from the hollow and filled cavity embedded InGaN flip-chip LEDs. Insets: Current-voltage curves of the same devices and an optical image of the wafer-level devices. **d** Measured angular transmission spectrum of the hollow (left) and filled (right) cavity embedded sapphire substrates depicted in (**a**). The white dashed lines indicate *θ*_c_ between GaN and the sapphire interface. **e** Simulated electric field profiles of a plane wave with an incident angle of 45° at λ = 450 nm at the hollow (left) and filled (right) cavity embedded GaN-sapphire interface. **f** Schematics of integrated ray-wave optics simulation for HCs-embedded InGaN filp-chip LEDs. **g** Simulated far-field intensity distributions of the cavity embedded flip-chip GaN LEDs at various *∆n* values. **a**–**c** are reprinted with permission from ref. ^2^ Copyright © 2016 American Chemical Society. **d** and **g** are reprinted from ref. ^6^ and ref. ^15^ with permission © 2018 Optical Society of America

The development of high-performance III-V semiconductor LEDs has created a new lighting market. One of the key factors in the fabrication of LEDs with higher brightness and efficiency is the improvement of their outcoupling efficiencies, that is, their ability to steer light trapped within a high-*n* medium into a low-*n* background medium against TIRs. The most common strategy for improving the outcoupling efficiency of LEDs is the introduction of quasi-random or periodically patterned surfaces on a doped semiconductor layer^25^, reflector^26^, or substrate^2^. For example, commercial GaN-based flip-chip LEDs utilize a 3-µm-pitch periodic pattern at the interface between *n*-type doped GaN and a sapphire substrate (right, Fig. 5a). Although a patterned sapphire substrate (PSS) can serve as a dielectric grating to transfer the Bloch momentum to trapped photons, its outcoupling performance is restricted because the refractive indices of GaN and sapphire materials are fixed (right, Fig. 5a). To address this index barrier problem, Moon et al. fabricated the same 3-μm-pitch array of HCs on a sapphire substrate and achieved high-quality epitaxial GaN growth^2^ (left, Fig. 5a). An HC-embedded GaN wafer induces more vivid interference colours relative to its filled-cavity counterpart (Fig. 5b), which is indicative of its strong diffraction owing to a large Δ*n*. Consequently, HC-based InGaN LEDs outperform commercial PSS devices in terms of optical performance (Fig. 5c). Importantly, the ultrathin (80 nm) crystalline alumina shells that cover the HCs provide a GaN growth template that is as excellent as PSS, resulting in negligible electrical degradation (inset, Fig. 5c).

Measurements of photonic band dispersion (i.e. angular transmittance spectra) on the HC and PSS samples illustrate how a high-Δ*n* grating can yield enhanced outcoupling efficiency^6^ (Fig. 5d). The practical significance of photonic band dispersion was revealed as an accurate method for quantifying the outcoupling performance of a given grating. A side-by-side comparison of the results indicates that the HC sample has a greater average transmittance over angles higher than *θ*_TIR_ compared to the PSS sample, which explains the competitive advantage of the HC-based InGaN LEDs in terms of optical performance. Electromagnetic simulations provided the key insight that an HC structure interacts more strongly with an incoming plane wave (Fig. 5e). Snapshots of the electric field indicate that the array of HCs significantly perturbs the propagation direction of the incident plane wave. In other words, it acts as an optical disturbance for incident light, resulting in a marked phase distortion in the vicinity of each HC structure (Fig. 5e). For a HC-based outcoupler, both a dramatic change in the phase and a high outcoupling efficiency with respect to the angles higher than *θ*_TIR_ facilitate the implementation of directive light sources. To demonstrate this assertion, the far-field intensity distributions of 2D gratings embedded in InGaN flip-chip LEDs were obtained at a variety of ∆*n* values by conducting integrated ray-wave optics simulations (Fig. 5f)^15^. In general, the ray-optics simulation is inappropriate to interpret the interaction of light with wavelength-scale structures (i.e. diffraction). In comparison, the wave-optics simulation properly accounts for diffraction-related phenomena, but due to a problem with computation memory, it is fundamentally incapable of designing ‘macroscopic’ optical devices that contain diffractive elements. Therefore, wave-optics simulation cannot be used to determine the extraction efficiency for each escape route and the far-field intensity distribution for an actual-scale GaN-based LED with a grating outcoupler.

An integrated ray-tracing simulation was developed to solve these serious issues with conventional optics simulations^15^. In the integrated ray-wave optics simulation, all diffracting light is converted into multiple rays propagating in predetermined directions. The procedure of the integrated ray-optics simulation for a 2D-grating-embedded InGaN flip-chip LED with a 100-μm-thick sapphire substrate is shown in Fig. 5f. First, wave-optics simulations were conducted to determine all orders of forward and backward diffraction efficiencies for the 2D grating under consideration as a function of incident angles. Then, the 2D grating was represented as a diffuse surface in a ray-optics model. The far-field intensity distributions of 2D grating-embedded LEDs were obtained at a variety of ∆*n* values (Fig. 5g). The results show that the beam divergence is monotonically reduced by increasing ∆*n*, owing to the improved extraction of light through the top side of the sapphire substrate as a result of diffraction. The PSS grating (∆*n* = 0.7) yields the maximum far-field amplitude at a polar angle of approximately 60°, with a local minimum at 0° to 30°. In comparison, the HC grating (∆*n* = 1.5) exhibits a Gaussian-like far-field distribution, which is useful for developing vertically directed light sources, including micro-LED-based near-eye displays in which substrate-side emission is not utilised.

#### Organic light-emitting diodes

**Fig. 6:**
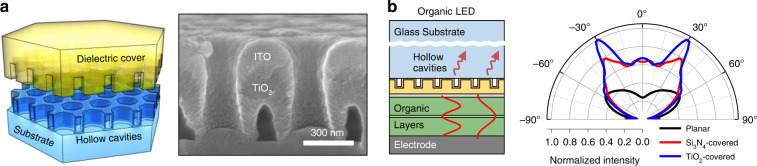
Submicron HC grating-embedded OLEDs. **a** (Left) Schematic of a hexagonally arranged submicron HC grating, which covered with a high-*n* dielectric layer. (Right) Cross-sectional SEM image of the fabricated HC grating-embedded film. **b** (Left) Schematic and (right) simulated far-field intensity distribution of a millimetre-scale (0.25 × 0.25 × 0.10 mm^3^) HC-embedded (with a Si_3_N_4_ or TiO_2_ cover) OLED device. A result of the planar organic LED device is plotted together. **a** and **b** are reprinted with permission from ref. ^29^ Copyright © 2021 Elsevier

HC gratings can be utilised in other semiconductors and organic LEDs (OLEDs) to enhance their outcoupling efficiencies. The active layer of OLEDs is covered with an optically thick and low-*n* glass substrate, such that the majority of the photons generated via electron-hole recombination are trapped and lost^27,28^. Therefore, the incorporation of an appropriately designed HC grating can efficiently extract tightly bound photons in such a waveguide configuration, thereby enhancing the optical performance of OLEDs (left, Fig. 6a). Recently, Kim et al. reported on a 500-nm-pitch hexagonal array of HCs covered with a TiO_2_ (*n* = 2.5) planarization layer (right, Fig. 6a)^29^. It is noteworthy that a ‘submicron’ HC grating is more effective for extracting light trapped in OLEDs because their active layer has a relatively low *n* (typically, *n* ~ 1.7) (left, Fig. 6b). The fabrication technology of HC arrays, as shown in Fig. 4c, can be readily extended to the submicron scale with high-resolution lithography systems (e.g. nanoimprint, stepper, etc.). The TiO_2_ planarization layer allows for a large Δ*n* to amplify the diffraction strength of the HC grating. Integrated ray-wave simulations of millimetre-scale OLEDs demonstrate that the TiO_2_-covered HC grating improves its outcoupling efficiency with reduced beam divergence because it steers horizontally guided photons into the ambient medium via the top surface of a glass substrate (right, Fig. 6b). As previously indicated, the addition of an HC grating to inorganic GaN-based LEDs also leads to a narrowing of the emission divergence (Fig. 5f).

### Metalenses

**Fig. 7:**
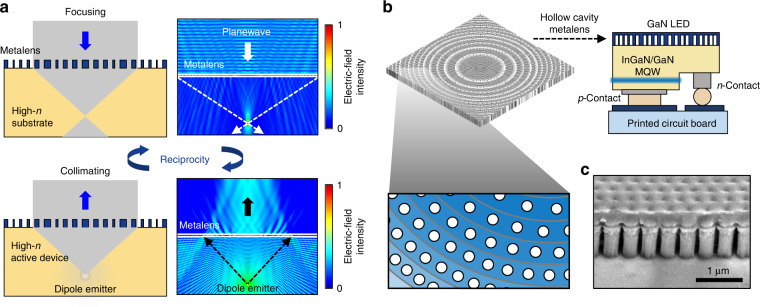
HC metalens integrated LEDs. **a** Schematic of the HC metalens integrated active devices based on the reciprocity principle. **b** (Left, top panel) Schematic drawing of HC metalens. (Left, bottom panel) Magnified image indicates a gradually tuned refractive index by arranging HCs. (Right) Schematic of the HC metalens integrated flip-chip InGaN LED. **c** Cross-sectional SEM image of the HC metalens integrated onto the LED device depicted in (**b**)

Another good application in terms of active devices using regularly arranged HCs is the development of metalens-integrated LEDs. A metalens is a virtually flat optical system composed of multiple subwavelength units with engineered phase retardation to mould the wavefront of scattered light^30–32^. Traditionally, metalenses have been employed to redirect incoming photons in free space to a focal point, making them highly competitive with bulk geometric lenses and refractive/diffractive Fresnel lenses^33,34^. When metalenses are monolithically incorporated into LEDs, new potential applications may emerge^35,36^. When the location of an active layer is coordinated at the focal point of an integrated metalens, the spherical waves generated by the active layer can be transformed into plane waves, which is a result of reciprocity^37^ (Fig. 7a). The integrated metalens can negate TIRs and shape the distribution of the emitted light, thereby contributing to the improvement of the outcoupling efficiency. Consequently, metalens -integrated LEDs are directional, efficient and compact light sources, which are of particular importance for augmented reality and virtual reality displays^26,38^. Most metalenses employ ‘high-*n*’ nanostructured units to cover the full range of 2π phase retardation with minimal plasma etching (Fig. 7b). Nonetheless, the manufacture of low-cost metalenses has proven to be difficult, and large-scale metalenses will exacerbate this problem. High-*n* dielectric-coated HCs, however, behave as similar phase retarders, and are more scalable and cost-effective to manufacture because of the absence of plasma etching (Fig. 7c). In addition, given that the fabrication temperature can be maintained low (<100 °C) throughout the entire process, as shown in Fig. 4c, incorporating an HC-based metalens into a working LED does not degrade the device characteristics.

## Light absorption devices

### Light trapping antennas

**Fig. 8:**
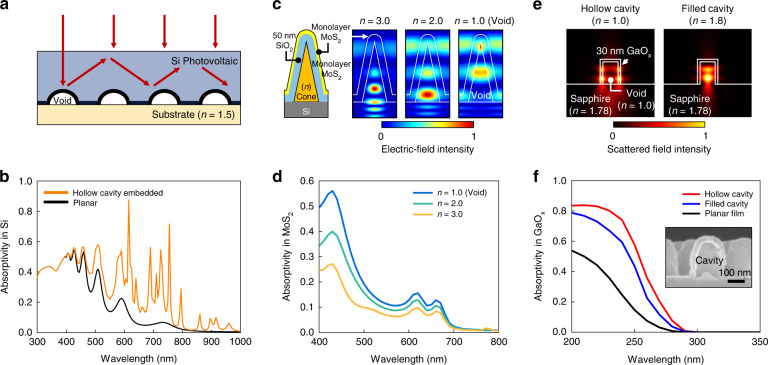
HC assisted absorption in ultrathin films. **a** Schematic illustrating the conversion of a normally incident plane wave into horizontally propagating waveguide modes by the introduction of embedded HCs. **b** Simulated absorption spectra of crystalline Si thin films (300 nm in thickness) with and without embedded 2D HCs. The pitch and diameter of the HCs are 500 and 350 nm, respectively. **c** (Left) Schematic and (right) electric field intensity distributions for the hexagonally arranged HC antenna (i.e. 0.7-nm-thick MoS_2_ coated nanocone array (300 nm in pitch)) with various *n* (i.e. *n* = 3.0, 2.0 and 1.0) at λ = 532 nm. The simulated structural parameters of the nanocone are 415 nm in diameter and 170 nm in height, respectively. **d** Simulated absorptivity spectra at the 0.7-nm-thick MoS_2_ monolayer of HC-antenna in c with various *n* (i.e. *n* = 3.0, 2.0 and 1.0). **e** Scattering field intensity distributions of the hollow (left) and filled (right) cavity-based 30-nm-thick GaO_x_ film. **f** Simulated absorptivity spectra at the 30-nm-thick GaO_x_ for the structures in (**c**). For comparison, a 30-nm-thick planar GaO_x_ film is also plotted. Inset: SEM image of the GaO_x_ coated HC array. The electric field distributions in (**c**) are reprinted from ref. ^42^ Copyright © 2018 The Royal Society of Chemistry

The strong scattering and diffraction of light induced by HCs and their arrays can enhance the optical performance of photon absorption devices, such as photovoltaics, solar absorbers and photodetectors. In principle, the introduction of a grating contributes to the improvement of absorption efficiency by (i) reducing the surface reflectance, for which a grating serves as a graded-index film^39^ and (ii) augmenting volumetric absorption, for which a grating converts incident light in free space into horizontally propagating guided light^23^. Owing to its large Δ*n*, the array of HCs embedded in a photovoltaic medium can be a good mode converter if appropriately designed, thereby dramatically increasing the absorption efficiency. A periodic pattern in a high-*n* thin film on a low-*n* substrate can convert incoming light into waveguide modes, which effectively arises with the use of a large Δ*n* pattern such as an array of HCs (Fig. 8a). Figure 8b shows simulated absorptivity spectra of crystalline Si thin films (300 nm in thickness) with and without embedded 2D HCs. The simulated data reveal that the HCs-embedded Si thin film creates multiple absorption peaks that are assigned to various orders of waveguide modes^40^. The emergence of the new waveguide modes finally leads to broadband absorption that is essential to the development of high-efficiency solar cells.

In addition to this functionality, individual HCs can act as localised light-trapping antennas when wrapped with a high-*n* absorption material (left, Fig. 8c). This strategy is particularly useful for the implementation of ultrathin photodetectors, including 2D transition metal dichalcogenide (TMDC) devices. A MoS_2_ monolayer is a well-known 2D TMDC material with a direct bandgap, but its absorptivity is inherently poor owing to its atomic thickness^41–43^. As a result, the 2D-TMDC-based photodetectors or photovoltaics can yield only up to ~10% absorptivity or ~1% power conversion efficiency^44^. Thus, to further improve the light absorption efficiency, light-trapping strategies have been investigated, including Fabry-Pérot cavities^42,45^, photonic crystals^46,47^ and plasmonic antennas^48,49^. HCs can funnel incoming light into their absorptive shell region, thereby serving as an effective light trapping platform for ultrathin absorbers. The simulation results clearly illustrate that the HC (*n* = 1.0) antenna gathers incident light most efficiently, even beyond its projected area, thus assuring strong light-matter interactions with the MoS_2_ monolayer. Consequently, the absoptivity of the MoS_2_ monolayer is greatly improved over the entire visible spectrum (Fig. 8d).

The same strategy can be adopted to develop high-efficiency amorphous gallium oxide (GaO_X_)-based thin-film (30 nm or less in GaO_X_ thickness) photodetectors. To date, crystalline and amorphous gallium oxide materials have generated significant research interest owing to their wide bandgaps, exceeding 5 eV^50–52^. Therefore, they are used for the development of deep ultraviolet (DUV) photodetectors that must respond to light of wavelength <300 nm, while precluding the absorption of light of longer wavelengths, including the visible spectrum. DUV photodetectors are indispensable for missile tracking^53,54^, space communication^55,56^, water and air purification^57,58^ and the sensing of biological molecules (e.g. food-borne fungi)^59,60^. In particular, GaO_X_-based DUV photodetectors facilitate fast and sensitive responses and are compatible with any substrate; GaO_X_ thin films on polymer substrates enable the implementation of flexible DUV photodetectors^61^. Snapshots of the electric-field intensity of the hollow (*n* = 1.0) and sapphire-filled (*n* = 1.78) cavities wrapped with a 30-nm-thick GaO_X_ layer reveal that the incident light is tightly bound in the GaO_X_ layer only for the HC (Fig. 8e). Owing to the light trapping effect, a HC-based 30-nm-thick GaO_X_ film exhibits an absorptivity of >0.8 at 200 nm and an average absorptivity of 0.48 within the DUV band (200–300 nm), which outperforms the filled cavity and planar samples (Fig. 8f). The array of HCs with a diameter of 200 nm was fabricated using nanoimprint lithography for these GaO_X_ film studies (inset, Fig. 8f).

## Thermal radiation devices

### Thermal emitters for thermophotovoltaics

Thermophotovoltaics (TPVs) are thermal emission-harnessed energy devices. TPVs use low-bandgap (e.g. GaSb, InGaAs and InGaAsSb) photovoltaic cells to convert photon energy released by high-temperature (typically >800 °C) thermal emitters into electrical energy^62–64^. Thermal emitters require a nanophotonic strategy to match their emission spectrum to the spectral response of photovoltaic cells (e.g. 0.5–1.7 µm for a GaSb photovoltaic cell)^65,66^. An ideal thermal emitter features a stepwise spectrum in which its emissivity is unity only at the working wavelengths of the photovoltaic cells. Such wavelength-selective thermal emitters have been implemented based on optical resonators^67^, photonic crystals^68,69^, one-dimensional hyperbolic metamaterials^70–72^ and nonintuitive complex patterns^73^. Previous solutions were effective at regulating the spectrum, but they lacked the thermal stability that is required for continuous and steady operation at high temperatures. For example, Stelmakh et al. achieved wavelength-selective thermal radiation from 2D optical cavities composed of refractory metal (e.g. tantalum and tungsten). The spectrum of thermal radiation was readily tuned by modulating the diameter of the optical cavities^74^. However, thermally induced stress, the oxidation of metal surfaces and the diffusion of metal elements deteriorated the level of thermal radiation over time. Recently, Oh et al. developed cermet (i.e. carbon-ceria hybrid composites) based thermal emitters^75^. The fabricated structure exhibited marginal changes in its absorptivity spectra after a 48-h heating test at 1000 °C. However, the average absorptivity at the working wavelengths of the GaSb photovoltaic cell was limited to 0.5, which needs further improvement.

**Fig. 9:**
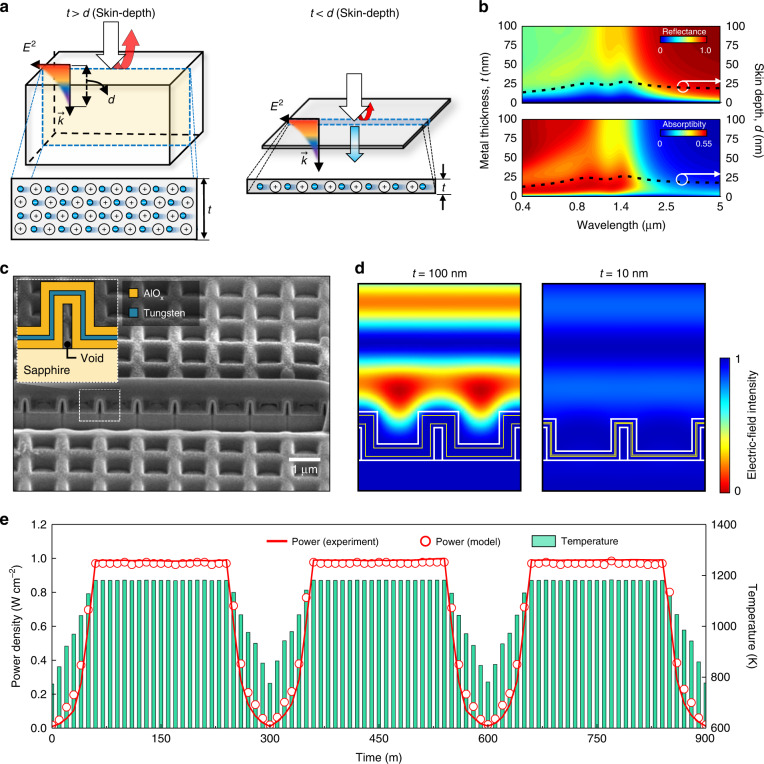
Sub-skin-depth thickness tungsten-based HC thermal emitters. **a** Schematic of the optical tunnelling effect through a sub-skin-depth (*t* **<** *d*) metal (right), contrasted with an optically thick (*t* **>** *d*) metal (left). **b** The surface plot represents the reflectance (top panel) and absorptivity (bottom panel) spectra of a tungsten film as a function of thickness (*t*). The dashed line depicts the skin depth (*d*) of the tungsten film. **c** Cross-sectional SEM images of the fabricated 2D array (950 nm in pitch) of HCs covered with alumina/tungsten/alumina (50/17/50 nm) multi-shells. (Inset) Schematic of the HC-based thermal emitter unit cell. The structural parameters in terms of the diameter and height of HC-based thermal emitter correspond to 400 nm and 770 nm, respectively. **d** Electric field intensity profiles for 100-nm- (left) and 10-nm-thick (right) HC-based thermal emitters at λ = 2.0 μm respectively. **e** Measured (solid line) and calculated (unfilled circles) TPV generated electric power during a repeated temperature cycling test between 500 K and 1200 K. A 17-nm-thick HC-based thermal emitter was used for this experiment. Reprinted with permission from ref. ^4^ Copyright © 2019 American Chemical Society

Recently, Cho et al. demonstrated photon-tunnelling-mediated thermal emission from an array of HCs covered with a deep subwavelength-thick (*t* = 10 nm) tungsten layer^4^. For bulk metal, the electric field of light is rapidly attenuated within the skin depth (*d*), which makes it highly reflective (i.e. less absorptive) (left, Fig. 9a). In comparison, for metals with *t* < *d*, light penetrates through them and the absorptivity improves (right, Fig. 9a). Electromagnetic simulations of tungsten films with various thicknesses show that they maintain reflectance less than 0.2 over the visible and near-infrared spectra (0.5–1.7 µm, the working spectrum of the GaSb cell) at *t* < *d* (top panel, Fig. 9b). The reduced reflectance enables the tungsten films with *t* < *d* to retain large absorptivity values over the same wavelengths (0.5–1.7 µm), despite their deep-subwavelength-thick nature (bottom panel, Fig. 9b). To improve the absorptivity of tungsten, a 2D array (950 nm in pitch) of HCs covered with alumina/tungsten/alumina (50/17/50 nm) multi-shells was fabricated using nanoimprinting lithography (Fig. 9c). Photon-tunnelling-mediated absorption/emission occurred in the structured tungsten, and the absorptivity of this metal was further augmented by optical resonance (Fig. 9d). Notably, the alumina shell and HC suppressed temperature-triggered oxidation and stress, respectively, thus improving thermal stability. As a result, the developed HC-based thermal emitter produced stable electric power during repeated temperature cycling tests between 500 K and 1200 K (Fig. 9e). The TPV generated electric power at discrete temperatures is in good agreement with the simulated data (unfilled circles, Fig. 9e) acquired from a one-dimensional energy conversion model based on the radiative heat transfer and diode equations. The TPV electric power density was approximately 1 W cm^−2^ at 1200 K, which is competitive with the performance of state-of-the-art TPVs at similar operation temperatures. The scalable and facile fabrication of structured metals leads to the development of thermal emitters with high-temperature stability and a tailored thermal radiation spectrum, which are essential for high-efficiency TPVs.

### On-chip antireflective radiative coolers

Mid-infrared (5–30 µm) photonics have inspired a surge of applications, including quantum cascade lasers^76^, molecule sensors^77^, thermal camouflage^78–82^ and radiative coolers^83–86^. In particular, research on RCs has attracted significant interest because it facilitates effective and passive (i.e. without energy consumption) heat dissipation channels^87^. RCs are classified into two categories: solar reflective^83,88,89^ and solar transmissive^5,87,90^, depending on whether the cooled object harnesses the solar energy. The ultimate goal of solar reflective RCs is to maintain the object temperature below the ambient temperature. To achieve sub-ambient radiative cooling, solar reflective RCs must be reflective in the solar spectrum (0.3–2.5 µm) and thermally black in the atmospheric window (8–13 µm)^11,91^. In contrast, to apply RCs to solar energy harvesting devices, such as solar cells, they must be transparent in the solar spectrum^5,90^. Visibly transparent but thermally absorptive/emissive materials, such as oxides (e.g. SiO_2_, TiO_2_, HfO_2_ and Al_2_O_3_) and various polymers (e.g. polydimethylsiloxane, polymethyl pentene and poly(methyl methacrylate)), are used in solar transmissive RCs. A few attempts have been made to apply RCs to solar energy devices^87,92^. For example, Zhu et al. reported a 6-µm-pitch square-lattice array of optical cavities with a 10 µm depth into a 500-μm-thick quartz wafer^92^. This patterned quartz wafer lowered the temperature of a Si wafer by 13 °C under one-sun illumination. In addition, Heo et al. constructed a 2D micron-grating (8.5 µm in pitch) with a 2 µm depth on a quartz wafer^87^. The fabricated structure cooled an integrated solar device (i.e. multijunction InGaP/GaAs/Ge photovoltaics) by 6 °C, thus yielding a 1.32% increase in the power conversion efficiency under sunlight of 900 W m^−2^. Although both approaches demonstrated effective cooling performance on a passive substrate or working optoelectronic device, they employed a dry-etching process that is not scalable and costly in fabrication. Moreover, they adopted an additional bonding process with an adhesive material to be integrated into devices, which could limit high-temperature applications such as concentrated photovoltaics.

**Fig. 10:**
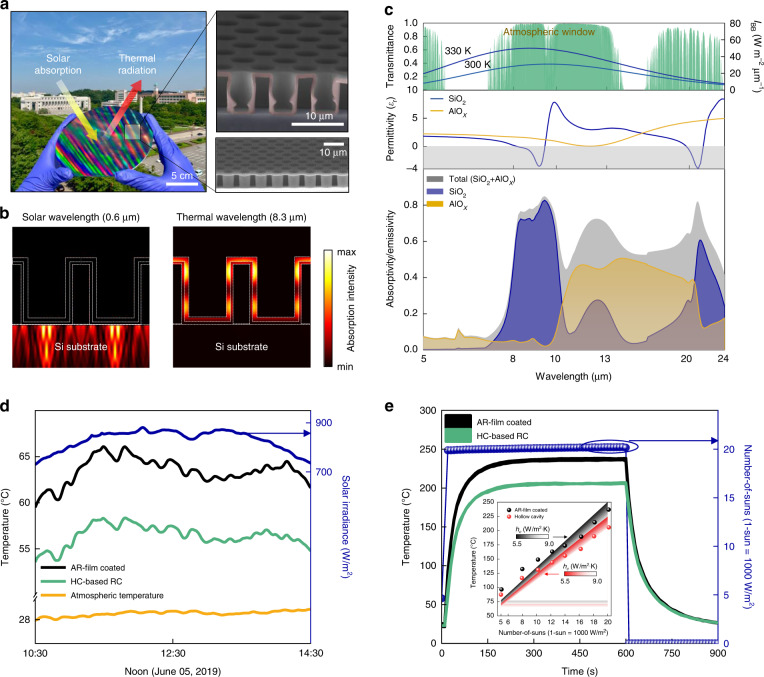
HC radiative coolers using oxide ceramics. **a** (Left) Conceptual camera and (right) SEM images of a hexagonal array (8 µm pitch) SiO_2_/AlO_x_ (500/300 nm) double-shell HC-based RC integrated into a Si wafer. The false-coloured area in the SEM image indicates the SiO_2_/AlO_x_ double-shell sustaining HCs. The structural parameters of the diameter and height for the HC-based RC are 6 µm and 8 µm, respectively. **b** Absorption intensity profiles of double-shell HC film at λ = 0.6 µm (left) and 8.3 μm (right). **c** (Bottom panel) Simulated absorptivity/emissivity spectra (λ = 5–24 μm) for double-shell HCs. (Middle panel) Permittivity real value of SiO_2_ and AlO_x_ materials and (top panel) transmittance of atmospheric window are represented. (Top panel) Both 300 K and 330 K blackbody spectra are plotted together. **d** Measured temporal temperature and solar irradiance changes for AR film-coated and HCs-based RC integrated Si wafer in the outdoor experiment. **e** Measured temporal temperature changes of Si wafers after solar simulator with concentrated twenty-sun intensity is activated/deactivated for the same structures in (**d**). Inset: Measured (filled circles) and simulated (shaded area) steady-state temperatures at various solar simulator intensities. The upper and lower boundaries in the shaded area represent different nonradiative coefficient conditions (*h*_*c*_ = 5.5–9.0 W m^−2^ K^−1^). Reprinted with permission from ref. ^5^ Copyright © 2020 American Chemical Society

Cho et al. recently reported on-chip solar transmissive RCs for application to solar cells and concentrated solar energy devices (Fig. 10a)^5^. Each HC works as an optical resonator; thus, its height must be larger than a few microns to effectively trap mid-infrared light. It should be noted that micron-depth plasma etching is expensive. Furthermore, the deposition of a few micron-thick oxide films typically causes fracturing, which could be exacerbated at high temperatures because of internal stress. However, the fabrication of HC-based RCs involves the use of submicron oxide films. Moreover, a depth of a few microns is readily achieved by selecting an appropriate photoresist with an appropriate coating thickness. These features account for the economic feasibility of HC-based RCs.

A hexagonal array (8 μm pitch) of SiO_2_/AlO_x_ (500/300 nm) double-shell HCs is antireflective in the solar spectrum and highly absorptive/emissive in the thermal radiation spectrum (Fig. 10b). The strong absorption of the subwavelength-thick oxide double shell originates from the same photon-tunnelling mechanism discussed in Fig. 9. To elucidate the excellent thermal emittance of the developed HC-based RC, the absorptivity of each oxide shell in the thermal-radiation spectrum was obtained (bottom panel, Fig. 10c). For comparison, the real parts of the permittivity (*ε*_r_) curves of the SiO_2_ and AlO_x_ materials are plotted in (middle panel, Fig. 10c). The simulation results convey two key points related to the large absorptivity/emissivity of the ultrathin double shell. First, the absorptivity of the outer SiO_2_ shell was significantly large near 9.1 μm and 21 μm, which is consistent with the local minima of *ε*_r_ of SiO_2_, originating from the different phonon-polariton resonances of SiO_2_. Second, the absorptivity spectrum is further broadened owing to the inner AlO_x_ shell; the *ε*_r_ of AlOx exhibited a relatively flat material dispersion with a local minimum at 11.8 μm. Consequently, the total absorptivity of the SiO_2_/AlO_x_ double-shell materials is larger than 0.6 at broad mid-infrared wavelengths (8−24 μm), which predominantly overlaps with the blackbody spectrum at 300 K. A proper figure of merit for each application should be established to characterise its performance; a figure of merit for RCs is an absorptivity/emissivity. Electromagnetic simulations prior to fabrication and characterization were carried out to determine the dependence of emissivity on the structural parameters of HCs. According to electromagnetic simulations, the diameter (*D*) and depth (*H*) of the HC-based RC have a significant impact on its emissivity^5^. The emissivity improves steadily with increasing *D* and *H*, revealing the importance of fabricating closely packed HCs with a few micron depths. The structural parameters of the fabricated HCs slightly deviate from the optimal ones. However, employing a thick (>7 μm) photoresist with a high-resolution mask aligner could result in an average emissivity that is greater than the current value, thus providing improved cooling performance.

The HC-based RC was used to conduct daytime cooling studies on Si wafers (Fig. 10d). An AlO_x_ (50 nm) film-coated Si wafer (labelled ‘antireflecting (AR) film-coated’ in Fig. 10d) was investigated for comparison, and exhibited the same amount of Si absorptivity as the HC sample while emitting negligible thermal radiation. During the day, the HC sample maintained lower temperatures than the reference, lowering the Si wafer temperature by 8 °C at a maximum solar irradiation of 881 W m^−2^, or approximately one-sun intensity. HC-embedded structures can minimise the thermal stress caused at high temperatures owing to their large vacancy fraction. After a 24 h heating test at high temperatures up to 800 °C under atmospheric pressure, the developed HC-based RC maintained its original thermal radiation spectrum and average Si absorptivity. Indoor cooling tests on Si wafers containing the developed HC film and the reference film were conducted using high-power (1000 W) xenon light and a Fresnel lens to determine the applicability of the concentrator photovoltaics (Fig. 10e). The temperature of both samples increased linearly when the light intensity increased from five to 20 sun. When both samples were heated at 200 °C under twenty-sun illumination, the HC sample exhibited a 31 °C temperature reduction compared to the reference film. Given that thermal radiation is amplified proportionate to the temperature to a power of four, according to the Stefan-Boltzmann equation, the temperature differential increases steadily (inset, Fig. 10e). It is worth noting that the blackbody spectrum of an object blueshifts as its temperature rises. Considering that the HC-based RC has a marginal emissivity at <8 μm, its average emissivity decreases as the solar intensity increases. At twenty-sun intensity, it maintains an average emissivity of approximately 0.6. Therefore, to apply the developed radiative coolers to solar energy devices operating at higher temperatures (e.g. >300 °C), more studies are required to investigate different dielectric shell materials with absorptivity/emissivity at shorter mid-infrared wavelengths (5−8 μm). Even at >8 µm, a Si_3_N_4_ material, for example, is relatively emissive. This study demonstrates that micron-depth HC films can be utilised to create thin-film mid-infrared emitters with readily tunable emission spectra by carefully choosing the shell materials.

## Perspectives

We have thus far discussed the diverse optical functionalities of HCs with properly designed morphology including their shell material and thickness. The array of HCs, for example, are unique diffracting elements that can be used in both organic and III–V semiconductor LEDs. These optical features aid in the recovery of photons trapped by TIRs, hence increasing the wall-plug efficiency of LEDs. The use of HC arrays can be further applied to light absorption devices such as solar cells and photodetectors to improve their photon-to-electron conversion efficiencies. Furthermore, individual HCs acting as optical resonators improve the performance of near-infrared thermophotovoltaics and mid-infrared radiative coolers, demonstrating their practical versatility. We emphasize that such an index engineering strategy for boosting diffraction and scattering strength is not restricted to the ‘optoelectronic devices explored herein, but will be acceptable to other devices. Understanding the underlying physics on the propagation of light and creating general design guidelines will enable a new horizon of optoelectronic devices that require high Δ*n* optical components.

HCs also offer exceptional thermal, electrical and mechanical characteristics. They can act as good thermal insulators, depending on the amount of interior voids^93,94^. Because HC-based composites have a low permittivity, they can be used to fabricate low-loss printed circuit boards^95^. In general, HC-embedded structures can help reduce weight and improve surface area, both of which are beneficial for drug delivery and catalysts^96^. The ability to withstand mechanical and thermal stresses may be advantageous in energy storage applications, particularly for lithium-ion batteries that are limited by volumetric changes during electrochemical cycling^97,98^. Because these features work independently (e.g., HC-based solar-transmissive RCs with mechanical flexibility, thermal insulation and high-temperature stability), HCs are regarded as a multifunctional and multiwavelength platform.

Fabrication of HCs and their arrays can be accomplished using both top-down and bottom-up methods. A self-templating method can be employed to implement HC particles of various sizes (e.g. from a few tens of nanometres to a few microns) in a scalable and low-cost manner because it avoids complicated processes such as core generation/removal and shell coating^99^. Synthesised HC particles can be mixed with printable pastes, thus creating new functionalities. They hold the promise of implementing three-dimensionally printed devices with reliable performance by providing antifouling and passive cooling functions with demanding heat and corrosion resistance.
